# Editorial: Functioning of individuals with cerebral palsy in the 21st century

**DOI:** 10.3389/fresc.2023.1205450

**Published:** 2023-06-20

**Authors:** Paula Silva de Carvalho Chagas, Hércules Ribeiro Leite

**Affiliations:** ^1^ Graduate Program in Rehabilitation Sciences and Physical and Functional Performance, Physical Therapy Faculty, Universidade Federal de Juiz de Fora, Juiz de Fora, Brazil; ^2^ Graduate Program in Rehabilitation Sciences, Department of Physical Therapy, Universidade Federal de Minas Gerais, Belo Horizonte, Brazil

**Keywords:** cerebral palsy, functioning, International Classification of Functioning, Disability and Health, children, rehabilitation

**Editorial on the Research Topic** Functioning of individuals with cerebral palsy in the 21st century

Cerebral Palsy (CP) is the most common motor disability in childhood, and it is a group of disorders that might affect in different levels a person’s ability to move, maintain balance, and posture (1).

The prevalence of CP in High-Income Countries (HICs) is 1.6 per 1,000 live births (2). However, data available from Low- and Middle-Income Countries (LMICs) indicate markedly higher birth prevalence than HICs (2). These children present a variety of possible patterns of mobility, communication, self-care, and participation across their lifespan, which might be influenced by different contextual experiences, such as personal factors and environmental barriers or facilitators at home, school, or community settings (3).

The goal of this research topic was to explore the diverse patterns of functioning that children with CP exhibit throughout their lifespan according to the International Classification of Functioning, Disability and Health (ICF) framework. This topic aimed to advance our understanding of how children and youth with CP live, how they are described, and how they are managed in the 21st century by promoting evidence and research in this area.

Significant changes have occurred in the field of CP management over the last few decades (4). The approach has shifted from focusing solely on impairments (i.e., treating brain function as a nonreactive organ and interventions targeting the secondary consequences of body function and structure) (5, 6) to an approach that recognizes the importance of (1) setting goals based on the client’s (i.e., child and family) preferences to promote activity and participation, with interventions focused on active practice of the entire task; (2) identifying the personal and environmental factors that might limit or facilitate the child/family chosen goals; (3) providing enjoyable and motivating intervention; (4) delivering interventions by parents in a natural environment and ensuring an adequate dose to achieve the established goals. Furthermore, it is important to highlight that all these good practices should place the child and caregivers at the core of the rehabilitation process, as decision-makers (7). We truly believe that this shift has been mainly promoted by the development of the ICF framework (5, 6, 8). Unfortunately, we have identified that this shift has not been implemented accordingly, especially in LMICs, where the focus is still on impairments, instead of promoting activity and participation (9, 10).

Papers published in this research topic highlight the new opportunities to “think outside the box” and promote functioning in this population in the 21st century. The paper by Longo et al. addressed the methods to evaluate the ingredients of therapy approaches (such as intervention dosage, principles, etc.) in early interventions for children with CP who are at highest risk of being non-ambulant (Gross Motor Function Classification System, GMFCS IV and V) and identify the ICF components and F-words addressed by these interventions. The F-words are a re-reading of the ICF framework which bring to life important concepts for child development [i.e., *fitness* (body structure and function), *functioning* (activity), *friends* (participation), *fun* (personal factors), *family* (environmental factors), and *future*]. From this perspective, these authors aim to provide a general menu of interventions, considering that this population often does not receive evidence-based interventions, especially in LMICs (11, 12).

The shared decision-making process in rehabilitation is important to identify which interventions are best suited and available to help individuals achieve their goals, while taking into account their current level of functioning (i.e., prognosis) (13). Functioning classification systems (such as those for self-care and mobility), have been used to identify the individual’s abilities (3). In their paper, Hou et al. demonstrated that the Mini-Manual Ability Classification System (Mini-MACS) is a valid and reliable tool for classifying manual ability in East Asian children with CP aged 1–4 years, and is also positively correlated with the Gross Motor Function Classification System. This information can inform parents and clinicians that a child’s level of mobility (e.g., ability to walk, run, and jump) may be closely related to their ability to perform self-care tasks (e.g., eating, dressing, and grooming) and that children with more restricted mobility may have reduced manual ability. This knowledge can help clinicians and caregivers to better understand a child’s abilities and limitations and to make informed decisions about which interventions to pursue to achieve the child’s chosen goals (3, 13).

Oliveira et al. reported two clinical cases of newborns with perinatal asphyxia who underwent therapeutic hypothermia and the follow-up of their motor development after being discharged from the hospital. Therapeutic hypothermia is a neuroprotective strategy used to reduce mortality and disability in children with hypoxic-ischemic encephalopathy caused by perinatal asphyxia (14). Both cases showed positive results and a good prognosis for motor development. This study presents an innovative therapy performed in an LMIC setting that may be a strategy to prevent neurologic sequelae, including CP, in newborns with perinatal asphyxia.

One other important goal during rehabilitation is to improve quality of life (QoL). The World Health Organization (WHO) defines QoL as a multidimensional construct that refers to individuals’ holistic perceptions of their wellbeing and their position in life, which is influenced by cultural, social, and environmental factors (12). However, sometimes children with CP have difficulties reporting about their own QoL. In such cases, we rely on proxy-reported measures. A study conducted Portugal by Vila-Nova et al. reported that, according to parent-reported measures, the QoL of children with CP is worse than that of their peers in 4 out of 10 domains measured by KIDSCREEN-12. This study highlights the importance of including QoL outcomes in our treatment goals for children with CP.

Finally, Knijnenburg et al. conducted a systematic review with the aim of identifying and examining neural reorganization of the sensory network in children with CP, including lesion type, somatotopic organization of the primary somatosensory area, and functional connectivity, in relation to sensory function. Understanding the extent and impact of sensory function on upper limb motor function is crucial for improving rehabilitation approaches and functional outcome. However, the review of 22 manuscripts highlighted the need for further research in this area.

Significant advances have been made in this century in understanding the functioning of CP. Figure 1 makes an analogy to present the main ingredients for therapy approaches that have been nourishing the life tree of people living with CP. We hope you all enjoy reading this research topic.

**Figure 1:**
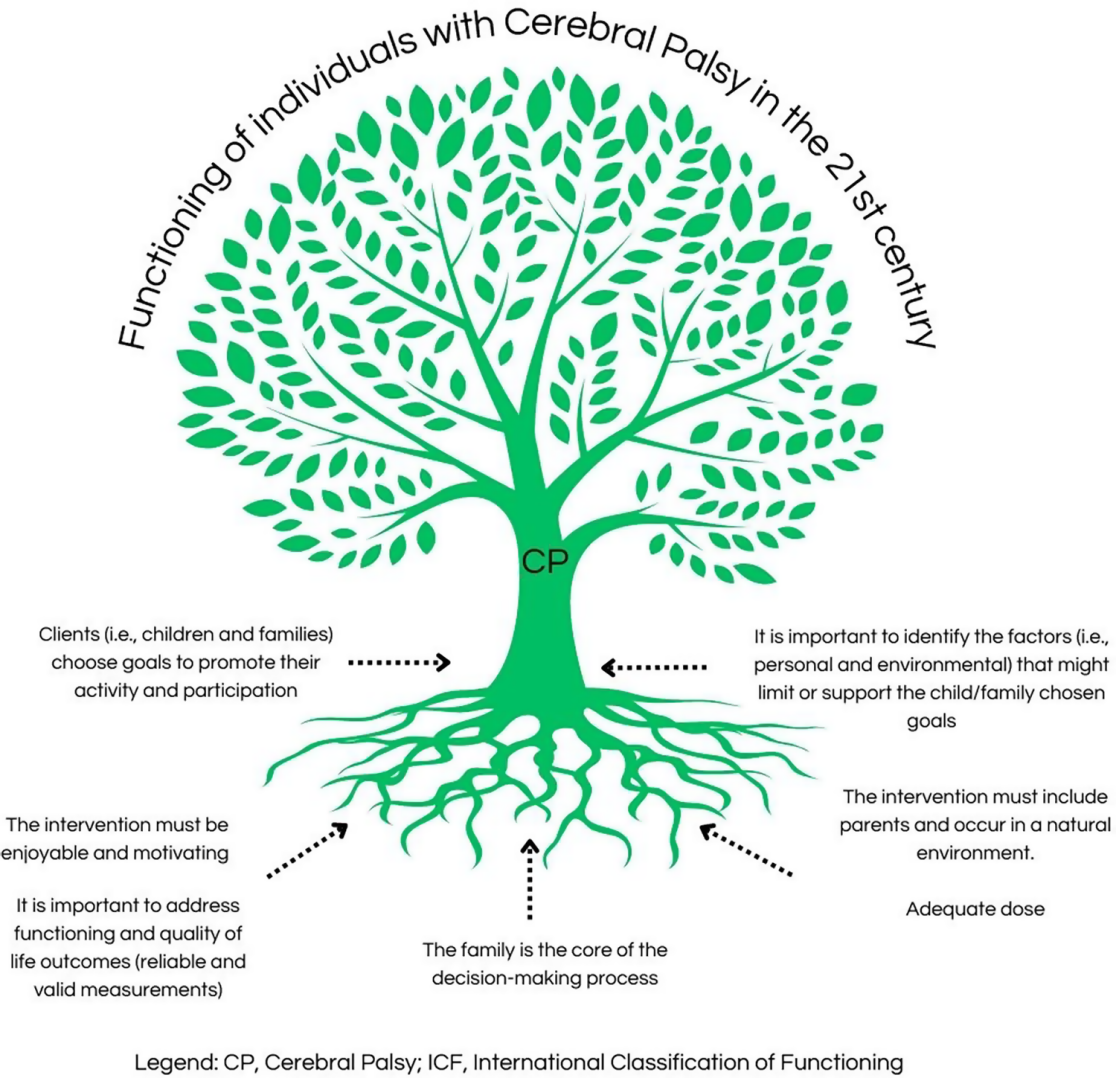
Main ingredients for therapy approaches that have been nourishing the life tree of CP people, based on Jackman et al. (7).

## References

[1] Rosenbaum P Paneth N Leviton A Goldstein M Bax M Damiano D A report: the definition and classification of cerebral palsy April 2006. Dev Med Child Neurol Suppl. (2007) 109:8–14. 17370477

[2] McIntyre S Goldsmith S Webb A Ehlinger V Hollung SJ McConnell K Global prevalence of cerebral palsy: a systematic analysis. Dev Med Child Neurol. (2022) 64(12):1494–506. 10.1111/dmcn.15346 35952356 PMC9804547

[3] Chagas PSC Magalhaes EDD Sousa Junior RR Romeros A Palisano RJ Leite HR Development of children, adolescents, and young adults with cerebral palsy according to the ICF: a scoping review. Dev Med Child Neurol. (2023) 65(6):745–753. 10.1111/dmcn.15484 36469744

[4] Novak I Morgan C Fahey M Finch-Edmondson M Galea C Hines A State of the evidence traffic lights 2019: systematic review of interventions for preventing and treating children with cerebral palsy. Curr Neurol Neurosci Rep. (2020) 20(2):3. 10.1007/s11910-020-1022-z 32086598 PMC7035308

[5] Carr JH Shepherd RB. The changing face of neurological rehabilitation. Braz J Phys Ther. (2006) 10(2):10. 10.1590/S1413-35552006000200003

[6] Leite HR Chagas PSC Rosenbaum P. Childhood disability: can people implement the F-words in low and middle-income countries—and how? Braz J Phys Ther. (2021) 25(1):1–3. 10.1016/j.bjpt.2020.07.006 32763088 PMC7817866

[7] Jackman M Sakzewski L Morgan C Boyd RN Brennan SE Langdon K Interventions to improve physical function for children and young people with cerebral palsy: international clinical practice guideline. Dev Med Child Neurol. (2022) 64(5):536–49. 10.1111/dmcn.15055 34549424

[8] Rosenbaum P Gorter JW. The ‘F-words’ in childhood disability: I swear this is how we should think!. Child Care Health Dev. (2012) 38(4):457–63. 10.1111/j.1365-2214.2011.01338.x 22040377

[9] Leite HR Jindal P Malek SA Rosenbaum P. Research on children with cerebral palsy in low- and middle-income countries. Pediatr Phys Ther. (2022) 34(4):551–5. 10.1097/PEP.0000000000000949 35960038

[10] Furtado MAS Ayupe KMA Christovao IS Sousa Junior RR Rosenbaum P Camargos ACR Physical therapy in children with cerebral palsy in Brazil: a scoping review. Dev Med Child Neurol. (2022) 64(5):550–60. 10.1111/dmcn.15067 34601719

[11] Bailes AF Greve K Long J Kurowski BG Vargus-Adams J Aronow B Describing the delivery of evidence-based physical therapy intervention to individuals with cerebral palsy. Pediatr Phys Ther. (2021) 33(2):65–72. 10.1097/PEP.0000000000000783 33770793 PMC10141519

[12] The World Health Organization Quality of Life assessment (WHOQOL): position paper from the World Health Organization. Soc Sci Med. (1995) 41(10):1403–9. 10.1016/0277-9536(95)00112-K 8560308

[13] Novak I Te Velde A Hines A Stanton E Mc Namara M Paton MCB Rehabilitation evidence-based decision-making: the READ model. Front Rehabil Sci. (2021) 2:726410. 10.3389/fresc.2021.726410 36188787 PMC9397823

[14] Abate BB Bimerew M Gebremichael B Mengesha Kassie A Kassaw M Gebremeskel T Effects of therapeutic hypothermia on death among asphyxiated neonates with hypoxic-ischemic encephalopathy: a systematic review and meta-analysis of randomized control trials. PLoS One. (2021) 16(2):e0247229. 10.1371/journal.pone.0247229 33630892 PMC7906350

